# Use of psychedelic treatments in psychiatric clinical practice: an EPA policy paper

**DOI:** 10.1192/j.eurpsy.2024.1806

**Published:** 2025-01-10

**Authors:** M. Destoop, P. Mohr, F. Butlen, P. Kéri, J. Samochowiec, L. De Picker, A. Fiorillo, K.P.C. Kuypers, G. Dom

**Affiliations:** 1 Collaborative Antwerp Psychiatric Research Institute (CAPRI), University of Antwerp, Antwerp, Belgium; 2 Multiversum Psychiatric Hospital, Boechout, Belgium; 3 Clinical Department, National Institute of Mental Health, Klecany, Czech Republic; 4 Third School of Medicine, Charles University, Prague, Czech Republic; 5 Office for Neurological and Psychiatric Disorders, European Medicines Agency, Amsterdam, The Netherlands; 6 Institut Gustave Roussy, Psycho-Oncology Unit, Interdisciplinary Department for the Organization of Patient Pathways, Cancer Campus, Grand Paris, France; 7 Université Paris-Saclay, UVSQ, Inserm, CESP, Villejuif, France; 8 Global Alliance of Mental Illness Advocacy Networks-Europe (GAMIAN-Europe), Brussels, Belgium; 9 Pomeranian Medical University in Szczecin, Szczecin, Poland; 10 University Psychiatric Hospital, Duffel, Belgium; 11 Department of Psychiatry, University of Campania Luigi Vanvitelli, Naples, Italy; 12 Department of Neuropsychology and Psychopharmacology, Faculty of Psychology and Neuroscience, Maastricht University, Maastricht, The Netherlands

**Keywords:** DMT, LSD, mental health, methodological challenges, psilocybin, Psychedelics, psychiatric disorders

## Abstract

**Background:**

Recent years show an exponential increased interest (“renaissance”) in the use of psychedelics for the treatment of mental disorders and broader. Some of these treatments, such as psilocybin for depression, are in the process of formal regulation by regulatory bodies in the US (FDA) and Europe (EMA), and as such on the brink of real-world implementation. In the slipstream of these developments increasing commercial initiatives are taking shape. The European Psychiatric Association (EPA) acknowledges both the therapeutic potential of psychedelic substances and the challenges for both research and clinical implementation. Steps need to be taken toward a well-balanced policy based upon sound scientific evidence and research, aiming at safe, ethical responsible integration of psychedelic therapy available for all patients who can potentially benefit.

**Methods:**

In this EPA policy paper, we highlight the potential benefits, and also the challenges of psychedelic treatments, which can be relevant for the future real-world implementation of these treatments.

**Results:**

In addition to an overview of the current evidence and hypotheses of working mechanisms of psychedelic treatment, this policy paper specifically highlights the importance of the psychosocial components of the treatment as well as the ethical and professional aspects playing a role in real-world implementation.

**Conclusions:**

Four recommendations are formulated for further research and clinical implementation.

## Introduction

Recent years show an exponentially growing interest (“renaissance”) in the use of classical psychedelics like psilocybin and LSD for the treatment of mental disorders [1]. This dynamic is driven on the one hand by the fast-growing evidence on the effectiveness and the relative safety of these treatments. On the other hand, psychiatry is confronted with the limitations of current pharmacological treatments. Non-response to medications for example is a significant problem with failure rates around 30% [2]. Taken together, pressures from and on professionals, patients, and families are increasing regarding the use of these “new” psychedelic medications. The situation is further complicated by a burgeoning public culture of non-medical substance use, fueled by media hype and commercialization. Legal and regulatory bodies, that is European Medicine Agency (EMA) and Food and Drug Administration (FDA), are seeking new paths, yet numerous questions persist.

The European Psychiatric Association (EPA) acknowledges both the therapeutic potential of psychedelic substances and the challenges for both research and clinical implementation. Steps need to be taken toward a well-balanced policy based upon sound scientific evidence and research, aiming at safe, ethical responsible integration of psychedelic therapy available for all patients who potentially can benefit.

In this EPA policy paper, we: 1) summarize the current body of evidence on the efficacy of psychedelic treatment, and main working hypotheses; 2) give an overview of the potential risks and adverse effects; 3) highlight challenges concerning research with these substances; 4) express potential ethical concerns; and 5) give advice on next steps for both research and clinical implementation.

Since the field of “psychedelics” is a broad pharmacological domain containing many different substances, we focus on classic psychedelic substances including psilocybin (present in ‘magic mushrooms’), lysergic acid diethylamide (LSD), and N,N-dimethyltryptamine (DMT, the major psychoactive molecule in ayahuasca).

## Current body of evidence

Providing a comprehensive overview of current research is beyond the scope of this policy paper. We refer to recent meta-analyses and reviews, cited below.

Rucker et al. (2016) conducted a systematic review involving 423 depressed patients, showing significant symptom improvement post-psychedelic treatment in a majority of participants (79.2%). These findings are supported by subsequent studies and meta-analyses [3–8], primarily focusing on single-dose psilocybin, which exhibits faster response rates compared to standard antidepressants like escitalopram, despite intermittent administration. Limited studies on anxiety and OCD patients show positive effects, though duration remains uncertain [9–10]. For terminally ill patients experiencing existential distress, psychedelics show promise based on a systematic review of 33 studies [11). Few studies address PTSD and classic psychedelics, but ongoing open-label trials with psilocybin and ayahuasca show promising results [12, 13]. A larger body of research including Phase 2 and Phase 3 trials with MDMA-assisted therapy in PTSD has been published over the last two decades, but this is outside the scope of this paper given our focus on traditional psychedelics [14]. Systematic reviews and meta-analyses on classical psychedelic treatment for substance use disorders yield mixed results, underscoring the need for larger, longer RCTs. Notably, a recent comprehensive study demonstrated substantial benefits in alcohol use when psilocybin is combined with psychotherapy compared to active placebo and psychotherapy alone [15]. Furthermore, it needs to be noted that some countries provide psychedelic-assisted treatment outside of studies, gain clinical experience in the use of psychedelics, and provide treatment guidance [16, 17].

Overall, although the majority of studies to date are small-scale and methodological issues remain, results suggest that psychedelics have beneficial effects on depression, anxiety, substance use, existential distress, and a variety of psychological domains, such as quality of life and well-being. However, more and larger studies are clearly needed.

## Adverse events

Including 44 studies on 598 unique patients, a recent systematic review explored adverse events (AEs) in clinical treatments with psychedelics [3]. Notably, in many studies, AEs were not systematically assessed or different adverse event assessment procedures were used. Despite these limitations, the authors concluded that treatments are overall well tolerated and AEs generally occur immediately following treatment while the patient is still under the more intensive care of the therapy team.

The most reported acute AEs include nausea, headaches, and anxiety for serotonergic psychedelics. Acute psychological adverse events (e.g., paranoid thoughts, feeling trapped, illusions, feeling abnormal, psychological discomfort) mostly resolve during sessions, but are sometimes severe.

Concerns have been raised about the addictive potential of psychedelics. However, it is noteworthy that classic psychedelics work on neurotransmitter systems unrelated to those targeted by drugs of abuse neither through dopaminergic effects, and as such are not addictive themselves [4].

Since we currently cannot predict individual responses and adverse reactions that may develop in the weeks or months following treatment, researchers and clinicians must perform longer-term follow-ups and report any untoward reactions. Suicidal ideation and suicidal behavior are rare and balanced with reports of decreased suicidality [5–7]. However, suicidality always constitutes a serious psychiatric emergency, which emphasizes the importance of investigating which patients are most at risk and how to best reduce the likelihood of their occurrence.

### Drug–drug interactions

Since psilocybin is currently moving towards real-world implementation and regulation, it is important to understand drug–drug interactions between psilocybin and psychiatric medications [8]. Indeed, previous studies on psilocybin-assisted therapy (AT) have generally excluded participants taking psychiatric medications or discontinued psychiatric medications before the administration of psilocybin. Recently, studies have shown the feasibility and safety of combining SSRI and psilocybin [9, 10]. More studies are needed here. Furthermore, most clinical trials excluded patients with co-morbid psychiatric or medical illnesses such as uncontrolled hypertension, cardiovascular disease, and liver disease. It remains unclear how to safely administer psilocybin-assisted psychotherapy (AT) to medically ill individuals, including individuals with liver damage that would affect drug metabolism.

### Working mechanisms hypotheses

Within the scope of this policy paper, a full overview of all research findings is not possible. For a more in-depth understanding, we refer to the many, excellent available reviews (e.g., [11, 12]).

Recent neuroscience theories propose that psychedelics disrupt neurobiological information-processing constraints, ultimately broadening the scope of perception, emotion, and cognition in a dose-dependent manner [13]. Overall, these models are hypotheses to describe neural processes and are subject to updates based on new findings.

The cortico-striatal thalamo-cortical (CSTC) model [18] and the relaxed beliefs under psychedelics (REBUS) model [19] emphasize the role of different subcortical structures (e.g., striatum and thalamus vs (para)hippocampus) in mediating psychedelic drug effects. The CSTC model proposes disturbed thalamocortical coupling leading to increased sensory information flow to the cortex [18], while the REBUS model suggests reduced top-down influence of higher-level cortical networks, leading to disturbed predictive coding processes [19]. A third theory, cortico-claustro-cortical (CCC), suggests psychedelics disrupt coupling between the claustrum (rich in serotonin (5-HT) 2A receptors) and the cortex [20].

In addition, psychedelics potentially alleviate anxiety and depression symptoms by reducing amygdala reactivity, modulating threat sensitivity, and decreasing default mode network (DMN) connectivity [21]. Their mechanism involves modulating glutamatergic neurotransmission, indirectly stimulating brain-derived neurotrophic factor (BDNF) linked to neurogenesis and neuroplasticity [22, 23]. Psychedelics may also reduce inflammation by modulating the immune system and normalizing pro-inflammatory cytokine levels associated with depression [24]. These processes likely contribute collectively to psychedelics’ therapeutic potential for anxiety and depression.

On an emotional-cognitive level, studies in healthy volunteers have demonstrated that psychedelics can acutely impact social processing, particularly emotional empathy. They increase emotional empathy, especially for positive emotions, potentially aiding social reconnection in addiction therapies. Additionally, they also reduce the recognition of negative emotions, fostering social approach behavior. Limited research suggests their potential impact on decision-making and social feedback integration in therapeutic settings. Studies investigating the long-term effects of psychedelics on social cognition and behavior remain scarce [24]. Next to changes in social behavior, longer-lasting changes in personality (e.g., increased openness) and mindfulness have been shown [25, 26].

## Methodological challenges in research

Several methodological challenges warrant caution, including expectancy, blinding, the therapeutic alliance and psychedelic experience of the therapist, and other biases; all these issues will be discussed below. For a more detailed discussion of those topics, we refer to for example, [27] or [28].

### Expectancy

Mertens et al [29] suggest offering the opportunity to every participant in a clinical trial to receive the ‘experimental treatment’ (here: psychedelics) after assessment of the primary endpoint to minimize nocebo-effects in comparator arms [29]. To underscore the importance, they refer to the study by Carhart-Harris et al where participants could either receive psilocybin or escitalopram [30]. The depression scores of the patients in the escitalopram group were lower than normally observed in the placebo arm in an escitalopram trial. They suggest that realizing they did not receive psilocybin, the disappointment caused them not to “believe” in the treatment (“expectancy”) and to not improve that strongly [29].

### Blinding

Several approaches have been used aiming to preserve the blinding of treatment, for example, substances that induced physiological effects, like niacin (vitamin B3) [31] or methylphenidate [32], or low doses of the psychedelic [29, 33], or tell the participants that they will receive either a placebo, hallucinogen, stimulant, or sedative drug (e.g. [34]). Although the latter has been used only in research with participants without mental disorders. Alternatively, patients can be asked to guess the treatment they received and on which this guess was based, but also what their expectation is [27].

### Effect of therapy and therapist

Psychedelic drug trials often integrate psychotherapy, complicating control of treatment variables. The therapeutic alliance formed between patient and practitioner, influential in treatment outcomes, might heighten the placebo response, potentially risking inflated evidence for psychedelics. Therapists being aware of treatment allocation could inadvertently deliver different therapies across groups, and integration therapies for the treatment group might not suit the control group, potentially causing confusion or discomfort for those on non-psychedelic placebos [35].

### Selective reporting bias

One of the biases mentioned in the literature is “selective reporting bias” as it is often unclear due to challenges in determining whether the reported outcomes were pre-planned [36]. Also, the self-selection bias is often highlighted in psychedelic research, posing challenges for representative ethnic and demographic sampling in mental health research, contributing to healthcare disparities [35]. The underrepresentation of minority scientists and therapists in this field further compounds this issue. Inadequate representation limits the generalizability of findings and could worsen existing inequities in healthcare. Explicit recruitment targets for diverse demographic groups in study protocols could address this. Additionally, the potential influence of experimenter biases and the fusion of investigators’ roles with advocacy in psychedelic science might raise doubts about the objectivity of reported data, potentially affecting the trust of future healthcare users [35].

### Pharmacovigilance and patient screening

Concerns about long-term negative outcomes (i.e., negative psychological responses lasting for at least 72 hours) persist despite no broad link to poor mental health in surveys [37]. Individual risk factors like psychosis susceptibility demand careful patient screening. Ongoing research seeks to pinpoint predictors of adverse responses, yet significant knowledge gaps remain. Identifying and safeguarding vulnerable patients before recommending psychedelic therapy is crucial, recognizing that not everyone is suited for these treatments [37]. Large clinical studies on long-term negative outcomes of approved psychotropic medication make it possible to select and prioritize contraindications, which is currently not possible due to a small number of qualitative studies on long-term outcomes after psychedelics [38]. Further and greater efforts need to be made to better prevent rare, but important, negative psychological responses to psychedelics [39].

### Single dosing versus repeated administration

Most research protocols with psilocybin have used a single-dosing scheme. Recently, Nutt et al. [40] suggested that this treatment regime will be repeated up to once more for psilocybin at no less than monthly intervals. However, more research is needed to evaluate the need (and for whom) for continuation of treatment.

## Psychosocial – psychotherapeutic aspects of the treatment

Psychedelics and therapy are often named “psychedelic-assisted (psycho)therapy.” In the earlier days of psychedelic research, there was also “psycholytic therapy,” using the psychedelic as an aid to psychotherapy, while psychedelic therapy was more focused on the experience, also using higher doses than the former therapy [41]. What we see today is a combination of both approaches, using the higher doses, used in psychedelic therapy, and having multiple integration sessions afterward to work through the material, though sometimes no extensive integration is done, more leaning towards the old psychedelic therapy model.

Overall, in clinical trials where psychedelics are used, we typically see two to three stages, including preparation, sessions with the psychedelic substance, and integration after psychedelic sessions take place; the latter is not standard in research protocols, but much of the psilocybin research, however, emphasized integration. The preparation phase informs individuals about what to expect, fosters rapport, and addresses questions. Following this, one or more inner-directed psychedelic sessions prompt individuals to delve inward. Integration sessions can follow, allowing reflection on the psychedelic experience and its potential for sustained cognitive and behavioral changes [42].

Therapeutic approaches range from basic support to integrating evidence-based psychotherapies like cognitive-behavioral therapy or acceptance and commitment therapy [43]. When used in preparation and integration sessions, evidence-based therapies, aim to enhance treatment efficacy by integrating therapeutic interventions and knowledge from established therapeutic approaches [43]. They offer advantages such as improved training for therapists, the potential for enhanced efficacy, and political legitimacy, they also introduce constraints on how therapeutic benefit is conceptualized in psychedelic-AT. This constraint may limit the broad range of therapeutic experiences that participants can have during sessions with the substance, potentially pressuring them to conform to a fixed set of treatment outcomes, risking harm, and undermining the participant’s understanding of their experience.

While psychedelic-AT in clinical trials is generally given on an individual basis, some recent studies have tried group therapy with psilocybin [44, 45], to investigate whether this option could maximize patient benefits and resource availability. Demonstrated as feasible, this approach could be explored more, to understand whether it adds therapeutic benefits while optimizing resource use.

### Ethical aspects and professional challenges

Given the speed the field is moving there is a pressing need to better understand how these treatments might be most ethically delivered. Indeed, reports of unethical conduct have already been reported [46, 47]. In addition to and despite evidence on an overall good safety profile of these treatments (5), critical voices remain highlighting that, although some patients respond well to these treatments, others might experience adverse reactions [48, 49].

Several ethical aspects warrant specific attention in the (future) use of psychedelics in psychiatric treatment. First, psychiatrists as professionals should avoid being swayed too heavily by the (media) headlines [46]. Indeed, despite the promising results, psychedelics are no wonder drugs, but the hype has gotten ahead of the science [4]. An evidence-based approach is essential when implementing psychedelic treatments in real-world psychiatry. In this sense, some authors caution against overemphasis on breakthrough therapy designations by regulatory authorities and advise against unwarranted enthusiasm or extrapolation from early trial successes, especially given the complexity of psychedelic therapy [37].

Second, psychedelic therapies demand specific informed consent due to their distinct effects [50, 51]. Enhanced consent methods are crucial, ensuring patients grasp the treatments’ nuances, considering intense consciousness shifts and potential changes in personality or beliefs. Enduring effects like increased openness or altered views should be disclosed pre-consent, allowing patients to opt-out if concerned. A rigorous consent process aligns with the “set and setting” approach, emphasizing the patient’s mindset and environment during therapy. Preparing patients before sessions, centered on informed consent, aims to ethically guide psychedelic therapy [37].

Third, one aspect to consider is the complexity of the patient-therapist interaction. Yet, there is no certification of a “psychedelic therapist” nor there is a consensus on what exactly is needed as psychosocial interventions within the context of psychedelic treatment. Most “psychedelic” sitters at this moment are not therapeutically trained and not governed by therapeutical ethical codes [47]. Indeed, as patients enter highly vulnerable and even regressed states with psychedelics, challenging aspects of the ordinary therapeutic relationship and processes are amplified [47]. While these treatments promote trust and openness, they can lead to overthrust and suggestibility, risking patient manipulation. Past ethical violations emphasize the need for rigorous ethical standards and specialized clinician training [37].

A final note of ethical concern is the rapid rise of interest by the private sector and the exponential influx of financial investments. This might create specific tensions, for example, it is easy to imagine how marketing hype could supplant evidence-based practice [52]. This warrants that patients, care providers, funding bodies, and researchers need a countervailing body of objective services research and economic analysis with a minimum of conflict of interest and a commitment to open science [53, 54].

### Training and education

The training landscape for psychedelic therapists is evolving with a focus on skills that impact outcomes, like empathy for altered states and immersive experiences. Current interventions vary from structured strategies to less rigid approaches due to constraints from pharmaceutical studies. Historically, therapist training was influenced by personal experiences with psychedelics [55], but this may change with a shift toward more scientific frameworks. While personal experiences could aid empathy during patient sessions, their significance is debated and needs further exploration. Training programs may acknowledge their value while aiming to balance their role, aligning psychedelic therapists’ skills with broader psychotherapy research [56].

A key emphasis in training is on integration skills, facilitating patients’ incorporation of psychedelic experiences into daily life. Combining third-wave cognitive-behavioral therapies with psychedelic treatments appears promising in connecting extraordinary experiences with practical, real-life changes [56].

### Next steps toward real-world healthcare implementation

Despite many questions remaining, based on the current (still limited) evidence and socio-legal dynamics in many countries, it is realistic to expect that (European) regulation and the possibility for a prescription for a limited (but growing) number of indications will be at hand soon. From a positive stance, when evidence on efficacy continues to accumulate, the implementation of psychedelic treatment might represent a paradigm shift in mental health care. This raises questions regarding both the societal impact and the professional healthcare as treatments are scaled up from research trials to real-world clinical practice [57].

Specifically for Europe, the European Medicine Agency (EMA) is in the process of evaluating the regulation of psilocybin. Within this context, the (3rd) revised draft Guideline on clinical investigation of medicinal products in the treatment of depression highlights the research and development for the repurposing of psychedelics [58]. It recommends that, as with all other antidepressants, randomized, double-blind, placebo-controlled, short-term trials are needed to establish a positive benefit/risk, as well as trials to confirm the maintenance of effect. In addition, it recommends starting a clinical investigation in a more severely affected population, such as patients with TRD [58, 59].

In parallel with the regulatory bodies, an important role is played by national and international scientific and medical associations. Indeed, these are key in shaping the context (guidance, protocol) within a real-world implementation of these new treatments. From this perspective, the EPA recommends that in future research and steps toward further clinical implementation:

1. The role of psychosocial and psychotherapeutic interventions needs to be clarified. Indeed, as summarized above, many questions remain about how the psychotherapy components of psychedelic AT produce meaningful benefits above and beyond the drug itself. We recommend testing the efficacy of adjunctive psychosocial treatments with a strong evidence base for the psychiatric indication of interest [60]. Based on growing evidence, protocols and guidance need to be developed regarding interventions during the preparation, drug administration, and integration phase of the treatment [59].
2. Given the complex ethical issues, there is a clear need to develop [60] standard guidance and protocols on how to deliver in an ethical and professionally responsible way these specific types of treatment [61].
3. Equity in research and clinical care delivery is advisable:
  - In addition to difficult-to-treat populations (e.g., treatment-resistant and partial responders), research needs to include a broader spectrum of populations to reflect real-world effectiveness. This includes considering differences in gender, socio-economic, race, and cultural background as well as patients with multiple (comorbid) disorders.
  - Psychedelic-AT must become available to everyone who can benefit. Now, it is an expensive treatment, thus there is a serious need to explore affordable delivery models across different healthcare contexts and different populations [47]. In addition, protocol development regulations and reimbursement rules need to enable equity in care delivery.
  - Health-economic analyses should be included in designing future studies.
4. Economic and commercial aspects should be taken into account. The last years have seen a rapid rise of interest by the private (for profit) sector. When interacting with these commercial entities, healthcare professionals need to be transparent and uphold strict ethical standards.

## Conclusion and call for action

The EPA is welcoming and recognizing the potential of psychedelic treatment as a promising field on the brink of real-world implementation. However, it warrants careful keeping to the evidence amidst the hyperinflated public and media hype and commercial interests. Further research is needed in larger trials to assess the efficacy and safety of both the pharmacologic drug aspects and the psychosocial therapeutic context. With pending regulations by the FDA and the EMA, the national and international medical and scientific bodies have to deliver broader clinical protocols and guidance. Safety, effectiveness, and accessibility of these new treatments are hallmark goals in developing these protocols. Some national associations have already produced standards (e.g., The Royal Australian and New Zealand College of Psychiatrists; RANZCP). However, given the specific European context governed by one EU regulating medicine body, there is a clear rationale to develop protocols and standards on a European level [62, 58]. These should guide the national psychiatric associations in the different European countries to develop their own, context-specific national protocols.

1. Evidence-based protocols need to be developed regarding interventions during the preparation, drug administration, psychotherapeutic interventions, and integration phase of the treatment.
2. Standard guidance on how to deliver in an ethical and professionally responsible way is indispensable.
3. Research needs to include a broader spectrum of populations to reflect real-world effectiveness and a health-economic analysis in order to make psychedelic-AT available to everyone who can benefit.
4. Healthcare professionals should be transparent about commercial aspects and uphold strict ethical standards.
